# Lumican effectively regulates the estrogen receptors-associated functional properties of breast cancer cells, expression of matrix effectors and epithelial-to-mesenchymal transition

**DOI:** 10.1038/srep45138

**Published:** 2017-03-23

**Authors:** Konstantina Karamanou, Marco Franchi, Zoi Piperigkou, Corinne Perreau, Francois-Xavier Maquart, Demitrios H. Vynios, Stéphane Brézillon

**Affiliations:** 1 Université de Reims Champagne Ardenne, Laboratoire de Biochimie Médicale et Biologie Moléculaire, Reims, France; 2 Biochemistry, Biochemical Analysis & Matrix Pathobiology Research Group, Laboratory of Biochemistry, Department of Chemistry, University of Patras, Patras, Greece; 3 CNRS UMR 7369, Matrice Extracellulaire et Dynamique Cellulaire, Reims, France; 4 Department for Life Quality Studies, University of Bologna, Rimini, Italy; 5 CHU de Reims, Laboratoire Central de Biochimie, Reims, France

## Abstract

Lumican is a small leucine-rich proteoglycan that has been shown to contribute in several physiological processes, but also to exert anticancer activity. On the other hand, it has been recently shown that knockdown of the estrogen receptor α (ERα) in low invasive MCF-7 (ERα+) breast cancer cells and the suppression of ERβ in highly aggressive MDA-MB-231 (ERβ+) cells significantly alter the functional properties of breast cancer cells and the gene expression profile of matrix macromolecules related to cancer progression and cell morphology. In this report, we evaluated the effects of lumican in respect to the ERs-associated breast cancer cell behaviour, before and after suppression of ERs, using scanning electron and confocal microscopies, qPCR and functional assays. Our data pinpointed that lumican significantly attenuated cell functional properties, including proliferation, migration and invasion. Furthermore, it modified cell morphology, inducing cell-cell junctions, evoked EMT/MET reprogramming and suppressed the expression of major matrix effectors (matrix metalloproteinases and EGFR) implicated in breast cancer progression. The effects of lumican were found to be related to the type of breast cancer cells and the ERα/β type. These data support the anticancer activity of lumican and open a new area for the pharmacological targeting of the invasive breast cancer.

Breast cancer is extensively studied as it constitutes about the one fourth of cancer cases among women^1^. A great hallmark of breast cancer is the absence or presence of estrogen receptors alpha and beta (ERα and ERβ). ERα-mediated signaling is the most important in breast cancer, as the 70% of cases are featured as ERα positive^2^. ERα is the major subtype in the mammary epithelium and therefore it constitutes a prognostic marker for breast cancer incidents. The endocrine resistance that ERα cells present is able to accelerate the growth of cancer cells, increases their aggressive behaviour and eventually provokes their transformation to differentiated mesenchymal cells, undergoing epithelial-to-mesenchymal transition (EMT), which is a key step toward cancer progression and metastasis^3^. Recently, it has been shown that ERα and ERβ are correlated with EMT, cell morphology and functional properties in breast cancer^4^,^5^. Moreover, ERs have been associated with the expression of extracellular matrix (ECM) macromolecules and therefore with the cell-matrix interactions and the organisation of tumour microenvironment^4^,^5^,^6^.

Extracellular matrix (ECM) is a dynamic network of macromolecules contributing to cell behaviour, gene expression and diverse functional properties^7^. Among the various ECM components, proteoglycans (PGs) are considered as multifunctional key effectors, as they are involved in numerous pathophysiological processes, including cancer^8^,^9^,^10^. PGs’ expression is remarkably altered during tumour development and growth and their modified exhibition on the tumour extracellular matrix and cancer cell membranes influences major cancer cell properties, such as cell proliferation, migration, invasion, angiogenesis and adhesion^11^. Small leucine-rich PGs (SLRPs) are among the most ubiquitously expressed class in the ECM. Their pericellular localization and the substitution of their core protein by one or more highly negative glycosaminoglycan (GAG) chains, enable the interactions of SLRPs with matrix effectors, such as cytokines, growth factors and cell surface receptors. These interactions lead to the modification of fundamental cell functional properties, such as migration, apoptosis, autophagy, angiogenesis, and metastatic potential of cancer cells^12^,^13^,^14^.

Lumican, a class II SLRP, contains a plethora of tyrosine sulphate residues at the N-terminus, whereas its protein core is substituted with the GAG keratan sulphate chain and polylactosamine. The human lumican gene encodes a protein of 338 amino acids^15^. Regarding ECM remodelling, lumican is involved in the inhibition of cancer invasion and metastasis, in the suppression of cell proliferation, in the inhibition of angiogenesis and in the inflammatory response^16^,^17^,^18^,^19^,^20^,^21^,^22^. Lumican is also a key regulator of the tumour matrix organisation and the cancer cell-matrix interactions due to its effects on collagen fibrillogenesis and degradation, binding to cell membrane integrins and receptors and eventually modulation downstream signalling pathways, as well as regulation of tumour cell functions^23^. Lumican is referred as both positively and negatively correlated with the tumour progression, as it markedly increases in the stroma of breast carcinomas^24^ and is highly expressed in melanomas^25^. Lumican also contributes to the regulation of the development of lung metastasis^25^.

In respect to breast cancer, low expression of lumican is correlated with the patients’ poor outcome^26^. The lower expression of lumican might be explained by the rapid progression of this tumour, but also by its low percentage of viability. Notably, the overexpression of lumican refers to the fibroblasts adjacent to cancer cells, and not to the cancer cells themselves^19^,^27^,^28^. Additionally, these increased levels of lumican correlate directly with increased tumourigenesis, lower levels of expression of ERs, but also the young age of patients.

Taking into consideration the importance of the expression of ERs and lumican in breast cancer cell properties and tumour progression and, in addition, the fact that lumican, due to its potent anticancer effect, may be a useful novel pharmaceutical agent for cancer targeting, we evaluated its effects in breast cancer cells of different ERα/β status. Specifically, we evaluated the effects of lumican on various types of breast cancer cells; one positive for ERα (MCF-7, low invasive) and another positive for the ERβ (MDA-MB-231, highly invasive) before and after knockdown of these ERs, respectively. It is worth noticing that lumican was found not only to significantly affect cell properties such as proliferation, migration and invasion, but also cell surface receptors, matrix macromolecules implicated in breast cancer progression and EMT markers. Our findings suggest that the effect of lumican is closely related to the ER type. To the best of our knowledge, this is referred for the first time in the literature. These findings are promising and could be potentially applied for designing novel pharmaceutical agents for breast cancer therapy.

## Results

### Evaluation of lumican effects on cell morphology of MCF-7/c and MCF-7/SP10+ breast cancer cells

**Figure 1:**
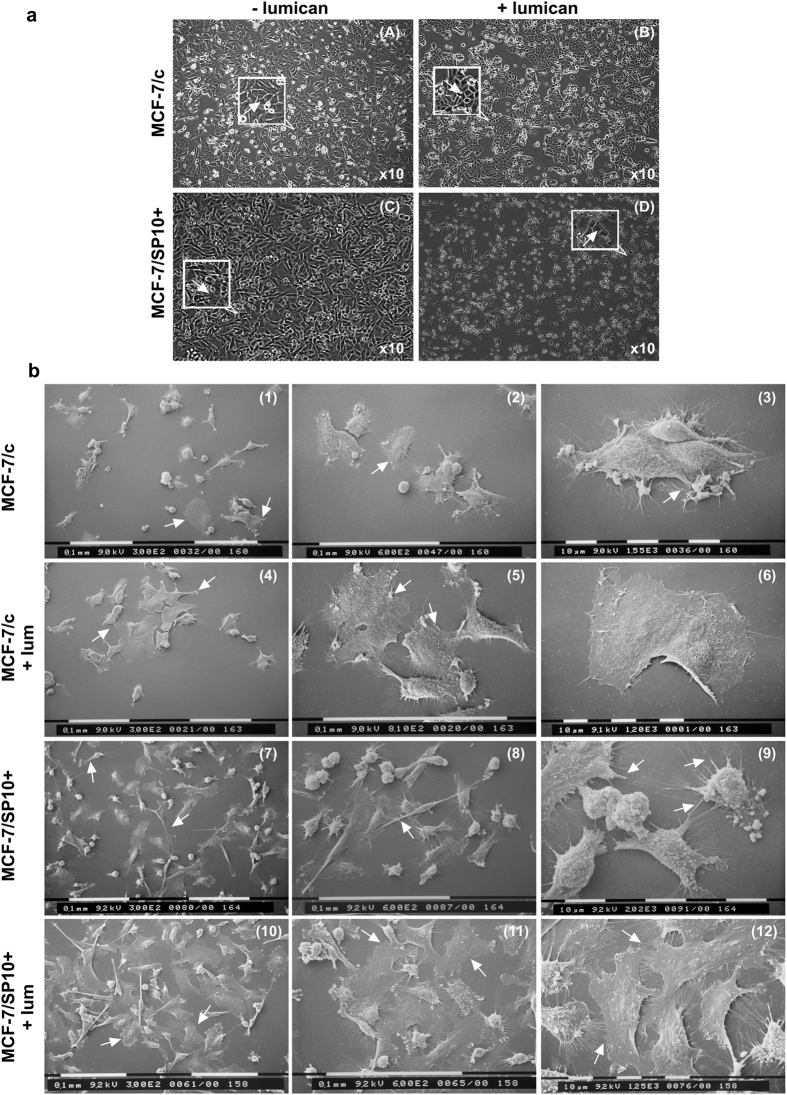
Evaluation of lumican effects on morphology of MCF-7/c and MCF-7/SP10+ breast cancer cells. (**a**) Monitoring cellular morphology of control MCF-7/c and MCF-7/SP10+ cells before and after treatment with lumican for 48 h by phase-contrast microscopy. MCF-7/c cells grew as closely packed colonies forming sheet-like monolayers (arrow in insert) displaying epithelial phenotype (image A). Lumican induced cell accumulation (image B). Cells treated with lumican appeared more globular as compared to control MCF7/c (arrow in insert). ERα knockdown cells (MCF-7/SP10+) exhibited a clear mesenchymal, spindle-like phenotype (arrow in insert) with total loss of cell-junctions (image C). In the presence of lumican, MCF-7/SP10+ cells exhibited both stem shape and spindle-like morphology (image D). A tendency for increase in cell-cell junctions was also observed (arrow in insert). (**b**) Scanning electron microscopy observations of MCF-7/c cells show a cell population mostly including relatively small rounded cells: few of them appear as small and globular but many of them show an ovoid, often flattened shape (arrows) with morphological characteristics resembling epithelial cells (1, 2, 3). Some of these cells appear in tight contact even though they show cytoplasmic protrusions like filopodia and few lamellipodia (3). After treatment with lumican, MCF-7/c cells appear more grouped and more flattened (arrows) (4, 5), most of them exhibiting an ovoid and larger shape (6). MCF-7/SP10+ cells show both small globular and ovoid shape with filopodia and many lamellipodia (arrows). Moreover, elongated or spindle-like cells with mesenchymal morphology are detectable (7, 8, 9). MCF-7/SP10+ cells treated with lumican are closer to each other (arrows) and appear very flattened with a very wide cytoplasm (10, 11, 12). Scale bars (0.1 mm and 10 μm) are indicated in the images.

First, we evaluated the effect of lumican treatment on cell morphology of MCF-7/c and MCF-7/SP10+ breast cancer cells. The cellular morphology of breast cancer cells was monitored by phase-contrast microscopy before and after treatment with lumican for 48 h in serum-free conditions. As shown in Fig. 1a, MCF-7/c cells displayed the characteristic epithelial morphology, forming sheet-like monolayers, following the ability to form cell-cell junctions (image A), which provide them low metastatic potential. Treatment of MCF-7/c cells with lumican (image B) induced cell accumulation, whereas cells appeared more globular as compared to untreated MCF-7/c cells. It has been recently demonstrated that the knockdown of ERα in ERα(+) MCF-7 breast cancer cells (MCF-7/SP10+) induced profound morphological changes, including mesenchymal characteristics such as elongated shape, numerous cytoplasmic protrusions (filopodia and lamellipodia) and the ability to grow distant to each other (Fig. 1a, image C). These features provide them significantly increased invasive potential as compared to MCF-7/c cells^5^. Treatment of MCF7/SP10+ breast cancer cells with lumican induced a tendency to appear more globular and to form cell-cell junctions (Fig. 1a, image D). Scanning electron microscopy (SEM) revealed lumican-related cellular alterations in MCF-7/c cells and a mesenchymal-to-epithelial transition (MET) in MCF7/SP10+ cells (Fig. 1b). In particular, MCF-7/c cells displayed a heterogenic cell population mostly including relatively small rounded cells, few of them appearing as small and globular but many of them showing an ovoid, often flattened shape with morphological characteristics resembling epithelial cells (images 1, 2 and 3). Some of these cells appeared in tight contact but still showed cytoplasmic protrusions like filopodia and few lamellipodia. Treatment of MCF-7/c cells with lumican seemed to favour cell-cell junctions thus inducing more cell groups and increased the number of larger, ovoid and more flattened cells (images 4, 5 and 6). MCF-7/SP10+ cells showed both small globular and ovoid shape with filopodia but more numerous lamellipodia compared to MCF-7/c ones. In addition to these cells elongated or spindle-like cells with mesenchymal morphology were visible (images 7, 8 and 9). These data confirm the strong invasive and higher migratory capacities of MCF-7/SP10+ cells as compared to MCF-7/c. Treatment of MCF-7/SP10+ breast cancer cells with lumican affected the spindle-like morphology by reducing the characteristic mesenchymal morphology of these cells and increasing the number of cell-cell junctions. MCF-7/SP10+ cells treated with lumican were closer to each other and appeared very flattened and very large compared to untreated cells, indicating that lumican may exert anti-migratory and anti-metastatic properties (images 10, 11 and 12).

### Lumican induces significant alterations in the EMT program of MCF-7/c and MCF-7/SP10+ cells

**Figure 2:**
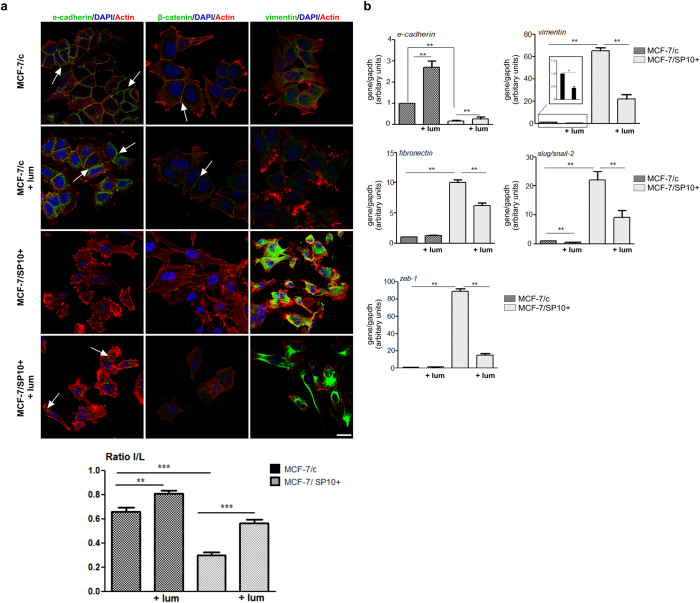
Lumican evokes MET reprogramming and significant alterations in the expression of EMT markers. (**a**) Confocal immunofluorescence imaging of E-cadherin, β- catenin and vimentin in MCF-7/c and MCF-7/SP10+ cells. E-cadherin was abundantly expressed at the membrane of cohesive MCF-7/c cells. In MCF-7/c cells incubated with lumican, E-cadherin appears at the cell membrane in cell-cell contacts, but it is also present in the cytoplasm. A slight cytoskeleton re-arrangement was also observed. The expression of E-cadherin was null in MCF-7/SP10+ cells but in the presence of lumican, E-cadherin immunolabelling can be detected (arrows). β-catenin labelling was detected all along the membrane of cohesive MCF-7/c cells. Lumican did not change β-catenin staining. This marker was poorly detected in MCF-7/SP10+ in absence or presence of lumican. In contrast to E-cadherin, vimentin was not detected in MCF-7/c in the absence or presence of lumican. Vimentin is highly expressed in MCF-7/SP10+ cells, showing that lumican did not affect vimentin’s expression. Scale bar: 10 μm. In the bottom panel, the ratio l (width)/L (length) of the MCF-7/c and MCF7/SP10+ cells before and after treatment with lumican are shown as to demonstrate the effects of lumican in the morphology of cells. (**b**) qPCR analysis of the EMT markers E-cadherin, vimentin, fibronectin, slug/snail-2 and zeb-1. Lumican significantly induced the gene expression of typical epithelial marker E-cadherin (2.7-times) in MCF-7/c cells, in agreement with the microscopic observation of increased cell-cell junctions and cellular accumulation. On the contrary, suppression of typical mesenchymal markers was observed. Vimentin and slug were decreased, in agreement with a more epithelial and globular morphology. Regarding MCF-7/SP10+ cells, a significant increase of the gene expression of E-cadherin was noted following treatment with lumican. In contrast, the mesenchymal markers were significantly suppressed. The significant reductions in the expression of vimentin (from 69.6 to 21), slug/snail-2 (from 22.8 to 8.5), and fibronectin (from 10.7 to 6.9) are in agreement with the more epithelial morphology and the potent MET observed following treatment of MCF-7/SP10+ cells with lumican. These data are also in agreement with the observed decrease in the number of filopodia (SEM imaging). Asterisks (*), (**) indicate statistically significant differences (*p* < 0.05 and *p* < 0.01, respectively).

The obtained data regarding the effects of lumican on breast cancer cell morphology generated the question whether the EMT program was affected by such treatment. Interestingly, as shown in Fig. 2a, confocal immunofluorescence analysis of MCF-7/c cells revealed that lumican increased the staining of the epithelial marker E-cadherin. Moreover, the treatment of MCF-7/c cells with lumican had no significant effect on the expression of β-catenin, whereas the low expression of vimentin was further reduced. The cytoskeleton formation of MCF-7/c breast cancer cells was not significantly affected by lumican treatment, as shown by F-actin staining (Fig. 2a). Notably, treatment of MCF-7/c cells with lumican significantly affected the gene expression levels of important EMT markers, as evaluated by qPCR (Fig. 2b). Specifically, the expression levels of the epithelial marker E-cadherin were significantly increased, whereas the mesenchymal marker slug/snail-2 was significantly downregulated as compared to the untreated cells. In respect to MCF-7/SP10+ cells, confocal microscopy confirmed that lumican relatively upregulated the protein levels of E-cadherin, whereas it reduced the protein levels of vimentin (Fig. 2a). These data were confirmed through real-time PCR analysis that revealed a significant increase in the expression levels of the epithelial marker E-cadherin. On the other hand, lumican significantly decreased the expression levels of important mesenchymal markers in MCF-7/SP10+ cells. Specifically, lumican suppressed the expression levels of vimentin, fibronectin, slug/snail-2 and zeb-1 (Fig. 2b). These data provide evidence that lumican evokes EMT/MET reprogramming in ERα knockdown cells, by suppressing the gene and protein levels of major EMT modulators in the mesenchymal, aggressive breast cancer cells.

The l/L ratio allowed us to characterize the EMT/MET processes affected by the knock-down of ERα (Fig. 2). The epithelial morphology of MCF-7/c was associated with a high width-to-length (l/L) ratio, while the l/L ratio was significantly decreased in MCF-7/SP10+ cells. The l/L ratio was affected by the presence of lumican (Fig. 2). MCF-7/c presented a slight increase of the epithelial phenotype. In addition, the effect of lumican was more drastic in MCF-7/SP10+ cells, endowing them with MET-like features.

### Lumican modulates the functional properties and expression profiles of major matrix effectors of MCF-7/c and MCF-7/SP10+ cells

**Figure 3:**
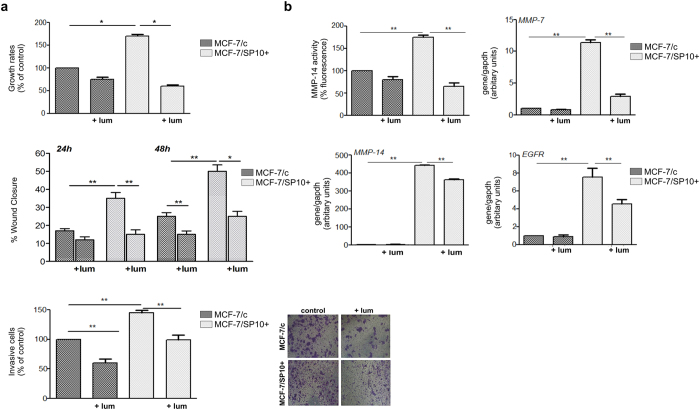
Lumican alters breast cancer cells functional properties and expression of matrix macromolecules implicated in cancer progression. (**a**) Cell functional properties including cell proliferation, migration and invasion were screened following cell cultures for 48 h in absence and presence of lumican. The proliferation of MCF-7/c was significantly lower (75%) than that of MCF-7/SP10+. Notably, lumican could decrease the proliferation of MCF-7/c and MCF-7/SP10+ by 20 and 70%, respectively. Moreover, lumican inhibited the migration of both cell types in a similar way. In respect to cell invasion, MCF-7/c cells exhibited significantly lower invasion potential than MCF-7/SP10+, whereas lumican significantly decreased the invasion of both cell lines. Each bar represents the mean of three independent experiments with SD values from triplicate samples. Asterisk (*) indicates statistically significant differences (p < 0.05). (**b**) Matrix macromolecules and cell receptors implicated in breast cancer progression have been evaluated before and after incubation with lumican. MMP-14 expression and activity was significantly higher in MCF-7/SP10+ cells compared to the control ones. Lumican suppressed the activity of both MCF-7/c and MCF-7/SP10+ cells, but the effect is more profound in MCF-7/SP10+ cells. MMP-7 followed a pattern similar to MMP-14. EGFR was highly expressed in MCF-7/SP10+ cells, but lumican induced its significant suppression.

It is well established that lumican regulates tumour cell functions in various cancer types and is strongly implicated in the cell-matrix interactions as well as in the matrix organisation^7^,^8^. Moreover, it has been reported that MCF-7/SP10+ cells exhibit 75% higher proliferation rates than MCF-7/c^5^. In order to evaluate the effect of lumican in the basal functional properties of MCF-7/c and MCF-7/SP10+ breast cancer cells, cell proliferation, migration and invasion assays were conducted. Cell proliferation was monitored with the wst-1 assay, in MCF-7/c and MCF-7/SP10+ cells after 48 h in absence and presence of lumican. Our results revealed that lumican decreased the proliferation rates of both MCF-7/c and MCF-7/SP10+ cells. Specifically, we observed that lumican slightly reduced the proliferation of MCF-7/c cells (ca 5%), whereas this effect was more profound in MCF-7/SP10+ cells (ca 70%) (Fig. 3a), suggesting that the effect of lumican on cell proliferation is related with the presence or absence of ERα. Cellular motility is regarded to be the central procedure of development and harmonized maintaining of life in all multicellular organisms. Malfunction of migration may increase the invasive or metastatic ability of cells. As shown in Fig. 3a, lumican significantly inhibited the migration of both MCF-7/c and MCF-7/SP10+ cells. At 24 and 48 h, the suppressive effect of lumican seemed notably important and reached 70-80%. These data were in agreement with the effects of lumican on cell morphology (Fig. 1a and b) as well as on EMT markers (Fig. 2a and b). It is important to notice that the similar inhibitory effects observed suggest that its effect on cell migration was not ERα-dependent. Invasion of cancer cells takes place because of their interactions with the neighbour cells of the tumour microenvironment and modified expression and secretion of macromolecules, such as matrix metalloproteinases, which cleave the ECM and favours cancer cells metastasis. The invasive ability of cancer cells is a process similar to the cell motility, with only one exception, the obligatory migration of the cell through a simulacrum of ECM^29^. MCF-7/c cells exhibit importantly lower invasive capacity than the MCF-7/SP10+. This suggests that cell invasiveness depends on the presence or absence of ERα, in agreement with recently published findings^5^. We observed that both MCF-7/c and MCF-7/SP10+ cells treated with lumican exhibit significantly reduced invasion levels, compared to the untreated cells, suggesting a clear anti-metastatic action. The similar patterns obtained upon treatment of ERα knockdown and ERα(+) cells with lumican suggest a non-correlation of lumican’s inhibitory role with the ERα status. It is well established that matrix macromolecules, such as membrane receptors and matrix degrading enzymes are strongly implicated in breast cancer progression. Therefore, we evaluated the effect of lumican on the expression profiles of major ECM components, in MCF-7/c and MCF-7/SP10+ breast cancer cells. As shown in Fig. 3b, lumican treatment of MCF-7/c cells did not affect significantly the expression levels of EGFR and several MMPs, including MMP-14 and MMP-7. On the other hand, lumican strongly downregulated the expression levels of these molecules in the highly aggressive MCF-7/SP10+ cells. Particularly, lumican significantly reduced the ERα knockdown-induced expression of MMP-7 and MMP-14 as well as the activity of the latter MMP. A significant reduction of the expression of EGFR in the aggressive MCF-7/SP10+ cells was also noted. These data suggest that lumican is a key inhibitor of the expression of critical players in breast cancer progression.

### Lumican modulates the morphology of MDA-MB-231 and shERβMDA-MB-231 cells

**Figure 4:**
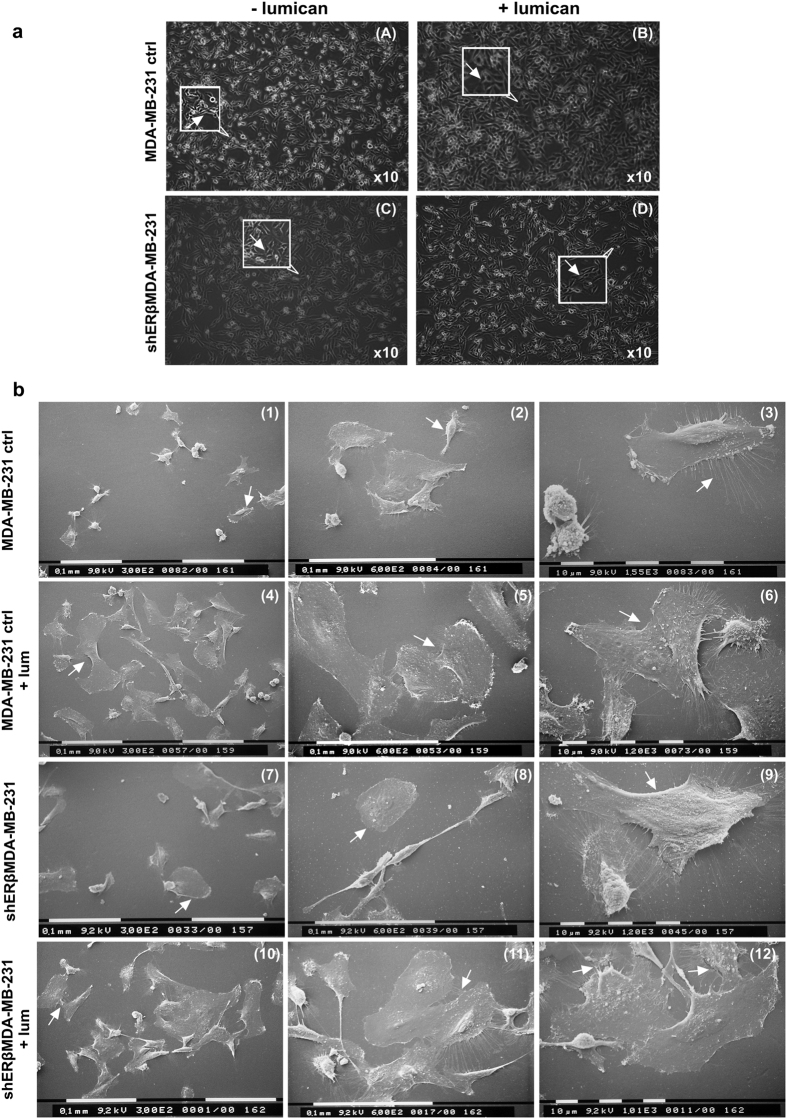
Lumican effects on morphology of MDA-MB-231 and shERβMDA-MB-231 breast cancer cells. (**a**) Screening of MDA-MB-231 and shERβMDA-MB-231 cells morphology by phase-contrast microscopy. MDA-MB-231 cells exhibit a typical mesenchymal, spindle-like morphology. Their elongated shape is indicated by an arrow in insert (image A). Upon their treatment with lumican, the co-existence of flattened and spindle-like shape was noted (arrow in insert of image B). Moreover, cell-cell contacts and cell accumulations were more evident than in the control cells. shERβMDA-MB-231 cells appear with altered cellular features compared with MDA-MB-231 cells. The shERβMDA-MB-231 cells displayed a tendency to demonstrate cell-cell contacts, an epithelial-like morphology with a more regular rounded outline (arrow in insert of image C). Incubation of shERβMDA-MB-231 with lumican enhanced the epithelial phenotype with cells appearing more adjacent to each other and with more visible and tight cell-cell contacts (arrow in insert of image D). (**b**) MDA-MB-231 cells observed at SEM show the mesenchymal characteristics represented by isolated and elongated cells (arrow) (1) with filopodia and lamellipodia (3), some globular rounded cells and few flattened ovoid ones (arrow) (2). MDA-MB-231 cells treated with lumican show an increase of flattened very large ovoid cells which appear more grouped, and show few filopodia and no lamellipodia (arrows) (4, 5, 6). Most of shERβMDA-MB-231 cells show a large and ovoid flattened shape with very few or almost absent filopodia and rare lamellipodia (arrows) (7, 8, 9). A rare spindle-like cell is also detectable (8). After lumican treatment most of shERβMDAMB-231 cells appear as epithelial like cells with a wide cytoplasm (11, 12) and tight cell-cell contacts (arrow) (10). Only few filopodia and rare lamellipodia are detectable (arrows) (11, 12). Scale bars (0,1 mm and 10 μm) are shown in the SEM images.

Recent study of our group demonstrated that the downregulation of ERβ in MDA-MB-231 breast cancer cells significantly reduced the aggressiveness of these cells through the inhibition of EMT accompanied by important morphological alterations^4^. Therefore, the effect of lumican on cell morphology of these two types of breast cancer cells was also evaluated. As shown in Fig. 4a, MDA-MB-231 cells display the typical mesenchymal, spindle-like morphology (image A), which supports their aggressive phenotype. These cells appeared as individual, elongated cells. Interestingly, treatment with lumican causes alterations of the morphology of these cells (image B). Interestingly, flattened and spindle-like cells could be co-observed and cell-cell contacts were evidently increased. It has been demonstrated that the suppression of ERβ in MDA-MB-231 cells increased the ability of these cells to form cell aggregates, with less elongated, epithelial-like cells^4^. These features were confirmed by phase-contrast microscopy since the treatment of shERβΜDA-MB-231 cells with lumican induced a more epithelial phenotype (Fig. 4a, images C and D). In order to further investigate these observations, the effects of lumican on the morphology of breast cancer cells were also investigated by SEM analysis. As shown in Fig. 4b, the mesenchymal characteristics of MDA-MB-231 cells could be confirmed by many cells with an elongated shape with filopodia and lamellipodia, a moderate number of globular rounded cells and very few flattened ovoid ones (Fig. 4b, images 1, 2 and 3). As shown in the images 4, 5 and 6 of Fig. 4b, the treatment of MDA-MB-231 cells with lumican increased the number of very large flattened ovoid cells with evident cell-cell contacts, so that cells appeared more grouped and showed less filopodia and rare lamellipodia. SEM analysis of shERβMDA-MB-231 cells showed that most of cells displayed a wide and ovoid flattened shape with very few or almost absent filopodia and rare lamellipodia (Fig. 4b, images 7, 8 and 9), even though some spindle-like cells were also detectable (image 8). When shERβMDA-MB-231 cells were treated with lumican for 48 h, most of cells still exhibited an epithelial pattern, but lumican seemed to favour an increase of cytoplasm and cell size as well as tight cell-cell contacts with a reduction of lamellipodia and sparingly of filopodia (images 10, 11 and 12).

### Lumican critically affects the expression levels of EMT markers in MDA-MB-231 and shERβMDA-MB-231 cells

**Figure 5:**
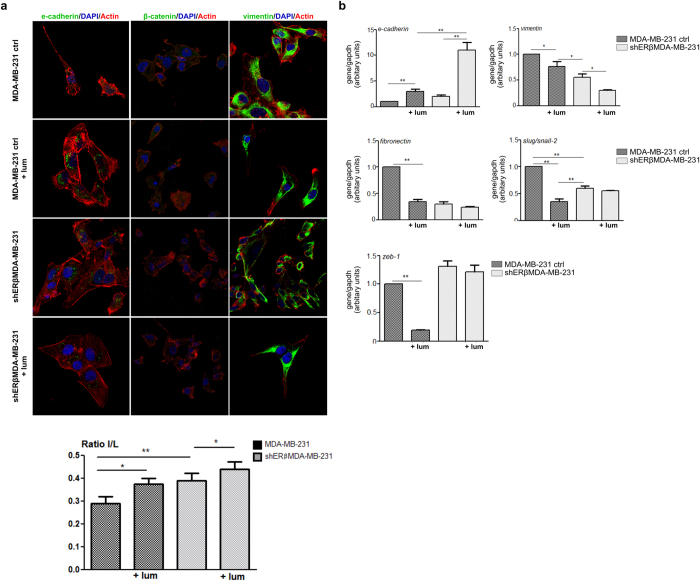
Lumican modifies the expression of typical EMT markers in MDA-MB-231 and shERβMDA-MB-231. (**a**) Confocal immunofluorescence microscopy of E-cadherin, β-catenin and vimentin. Vimentin was highly expressed in MDA-MB-231 non-treated and lumican-treated cells. Lumican induced slight actin cytoskeleton rearrangement. Vimentin was less expressed in shERβMDA-MB-231, and there were no profound alterations by the treatment with lumican. Ε-cadherin was poorly expressed in MDA-MB-231 control cells, but lumican increased its expression. In shERβMDA-MB-231, E-cadherin was found in traces and endowed an altered cell distribution. After lumican treatment, E-cadherin appeared still in traces. Interestingly, β-catenin was not expressed in MDA-MB-231 control cells, but lumican induced the expression of β-catenin in traces. Concerning, shERβMDA-MB-231, no significant changes were observed (bar, 10 μm). In the bottom panel, the ratio l (width)/L (length) of the MDA-MB-231 and shERβMDA-MB-231 cells before and after treatment with lumican are shown as to demonstrate the effects of lumican in the morphology of cells. (**b**) qPCR analysis of the EMT markers E-cadherin, vimentin, fibronectin, slug/snail-2 and zeb-1. After 48 h of incubation, lumican induced significant increase of the gene expression of the typical epithelial marker E-cadherin (*ca* 8-times) in shERβMDA-MB-231. In contrast, the mesenchymal marker expression is significantly inhibited by lumican after 48 h of incubation. Asterisks (*) and (**) indicate statistically significant differences (*p* < 0.05 and *p* < 0.01, respectively).

The observed changes induced by lumican in the morphology of MDA-MB-231 and shERβMDA-MB-231 cells prompted us to investigate the lumican effect on EMT. As shown in Fig. 5a, confocal microscopy revealed that MDA-MB-231 cells treated with lumican exhibited obviously increased protein levels of the epithelial marker E-cadherin, whereas it decreased vimentin protein levels. These results were confirmed with real-time PCR analysis (Fig. 5b). Moreover, lumican decreased the expression levels of the mesenchymal markers slug/snail-2 zeb1, vimentin and fibronectin and the epithelial marker E-cadherin in MDA-MB-231 breast cancer cells. These data suggest that lumican induced a potent MET pattern in MDA-MB-231 breast cancer cells. On the other hand, lumican treatment of shERβΜDA-MB-231 breast cancer cells revealed a slight increase of the expression levels of the epithelial marker E-cadherin, whereas it decreased that of the mesenchymal marker vimentin (Fig. 5a). No significant changes in β-catenin were noted. Real-time PCR confirmed the expression levels of E-cadherin and vimentin observed by confocal microscopy. The expression levels of fibronectin, slug/snail-2 and zeb1 were reduced but this effect was not statistically significant.

The EMT/MET processes were affected by the suppression of ERβ as shown by the l/L ratio (Fig. 5). The mesenchymal morphology of MDA-MB-231 cells was associated with a low l/L ratio, while it was significantly increased in shERβΜDA-MB-231 cells. The clear mesenchymal phenotype of MDA-MB-231 cells was significantly altered by the presence of lumican as demonstrated by the elevated l/L ratio turning them into a MET-like status (Fig. 5). In parallel, the pro-MET effect of lumican was also observed in shERβΜDA-MB-231 cells.

### Lumican modulates the functional properties of MDA-MB-231 and shERβMDA-MB-231 cells as well as the expression of major matrix components

**Figure 6:**
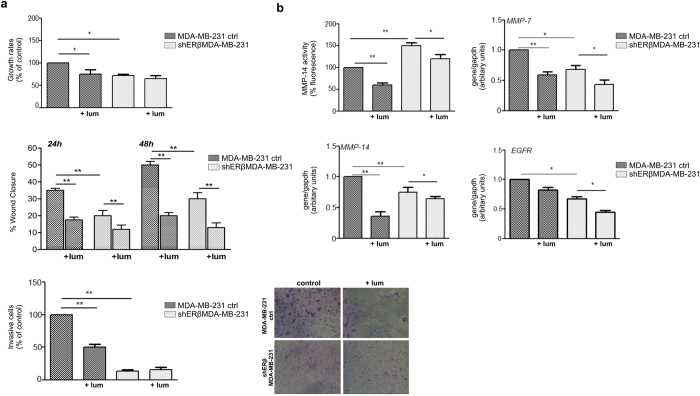
Effect of lumican in the functional properties of control MDA-MB-231 and shERβMDA-MB-231 and alterations induced in matrix macromolecules. (**a**) Cell proliferation, migration, and invasion were monitored in 48 h cell culture in the presence and absence of lumican. The lumican inhibitory effect on proliferation of MDA-MB-231 and shERβMDA-MB-231 cells was relatively low (20–25%). shERβMDA-MB-231 cells migrated in a different mode compared to the MDA-MB-231 control, suggesting a critical role for ERβ. Similarly with the MDA-MB-231 control cells, lumican strongly decreased the migration of shERβMDA-MB-231 cells.With regards to cell invasion, MDA-MB-231 cells exhibit significantly higher invasive potential than shERβMDA-MB-231. Lumican strongly decreased the invasion of MDA-MB-231 but had no effect on the invasive potential of shERβMDA-MB-231 cells. (**b**) MMP-14 activity is significantly lower in shERβMDA-MB-231 cells than in the control ones. Lumican suppressed MMP-14 activity of both MDA-MB-231 control cells and those with suppressed ERβ. Gene expression of MMP-14 was inhibited by lumican in MDA-MB-231 and shERβMDA-MB-231 cells. Decrease of MMP-7 gene expression in MDA-MB-231 and shERMDA-MB-231 was also observed in presence of lumican. In respect to matrix receptors, EGFR gene expression is altered between MDA-MB-231 and shERMDA-MB-231 cells. Lumican exhibits a similar inhibitory pattern in both cell types.

We investigated cell proliferation upon 48 h incubation with lumican. It has to be mentioned that the 24 h results are not displayed, as the effect was similar to 48 h. The lumican inhibitory effect on MDA-MB-231 and shERβMDA-MB-231 cells was relatively low (20–25%) and not related with the presence or not of ERβ (Fig. 6a). Moreover, lumican strongly decreased the migration of shERβMDA-MB-231 cells, which exhibited a profile similar to the control ones. Therefore, lumican’s inhibitory effect is similar for the control and ERβ suppressed cells suggesting that its effect on cell migration is not ERβ-dependent. Furthermore, MDA-MB-231 cells exhibited significantly higher invasion potential than shERβMDA-MB-231 and therefore the invasion depends on the ERβ cell status. We revealed that lumican strongly decreased the invasion capacity of MDA-MB-231, but did not affect the invasive potential of shERβMDA-MB-231 cells, indicating a role for ERβ in respect to lumican effect. This could also be attributed to the fact that the invasion index of shERβMDA-MB-231 cells is very low.

Breast cancer cell behaviour is highly affected by the matrix composition. Concerning the highly invasive MDA-MB-231 cells, the presence of lumican significantly reduced the proteolytic activity of MMP-14 as well as its expression levels (Fig. 6b). Moreover, it reduced the expression levels of MMP-7 and its presence did not significantly affect EGFR. Furthermore, shERβMDA-MB-231 breast cancer cells treated with lumican exhibited reduced proteolytic activity of MMP-14 and expression levels of MMP-7. The strongly reduced levels of EGFR upon ERβ suppression expression were further decreased by the presence of lumican in these cells. It should be noticed that, in contrast to MCF-7/c cells, the presence of lumican presented a similar profile of inhibition both in the suppressed and control cells, indicating not clear correlation with the expression of ERβ.

## Discussion

Cell morphology and especially epithelial-to-mesenchymal transition has been well correlated with the highly invasion potential of breast cancer cell^3^,^4^,^5^. ERs have been reported to play a crucial role in this process. Specifically, the knockdown of ERα in the epithelial and low invasive MCF-7 breast cancer cells leads to EMT and increases the aggressiveness of these cells (MCF-7/SP10+) that exhibit a significantly modified matrix expression pattern and different functional properties, i.e. higher migratory and invasive potential^5^. The obtained patterns following ERα knockdown are similar to those found in ERα-negative highly aggressive MDA-MB-231 cells which are ERβ-positive. On the other hand, suppression of ERβ in MDA-MB-231 cells attenuates the migratory and invasive profile and evokes a more epithelial phenotype^4^. These four cell lines could be therefore used as a model to evaluate the potential action of an anticancer agent in relation to the different ER status, the mesenchymal phenotype, the invasion potential and the modified expression of ECM macromolecules and cell receptors.

In this study, we evaluated the effects of lumican in the expression of matrix effectors, cell morphology and functional properties of MCF-7/SP10+ *vs* MCF-7/c and shERβ*MDA*-MB-231 vs MDA-MB-231 breast cancer cells. As mentioned above, the ER status is closely linked to the EMT program affecting crucial breast cancer cells properties. In this study, the obtained data concerning the mediation of breast cancer cell properties by lumican, clearly suggest significant alterations of the basal cell functional properties, such as proliferation, migration and aggressiveness. Notably, the anti-proliferative effect of lumican was evident in the highly proliferating ERα-negative MCF-7/SP10+ and MDA-MB-231 cells, whereas the inhibitory effect of lumican on cell migration and invasion did not depend on the presence of ERα. Moreover, lumican evokes interesting changes in cell morphology, affecting also the expression profiles of several ECM macromolecules.

The epithelial/mesenchymal features of cells were evaluated by phase-contrast, scanning electron and confocal microscopy, cell width-to-length ratio and expression of EMT markers (E-cadherin, fibronectin, vimentin and pro-EMT transcription factors such as slug and zeb-1). Regarding MCF-7 cells, microscopic and qPCR analyses revealed a clear epithelial morphology with apparent cell-cell junctions, high I/L ratio, and expression of E-cadherin in cellular periphery. Lumican significantly increased the membrane and intracellular staining of E-cadherin in MCF-7/c which may also indicate an increased neosynthesis of this marker and/or increased remodelling.This point has to be addressed in future studies. However, taking into consideration the significantly induced gene expression demonstrated by real time PCR, it is plausible to suggest an induction of the neosynthesis.

On the other hand, MCF-7/SP10+ cells appear to have elongated shape, low I/L ratio, absence of E-cadherin and numerous cytoplasmic protrusions, which endows them with significant higher invasive capacity. In lumican-treated MCF-7/SP10+ cells, the spindle-like morphology is affected (significant increase in the I/L ratio) and the cells present a potent MET as they look more flattened, with less filopodia and with increased expression of epithelial *vs* mesenchymal markers.

**Figure 7:**
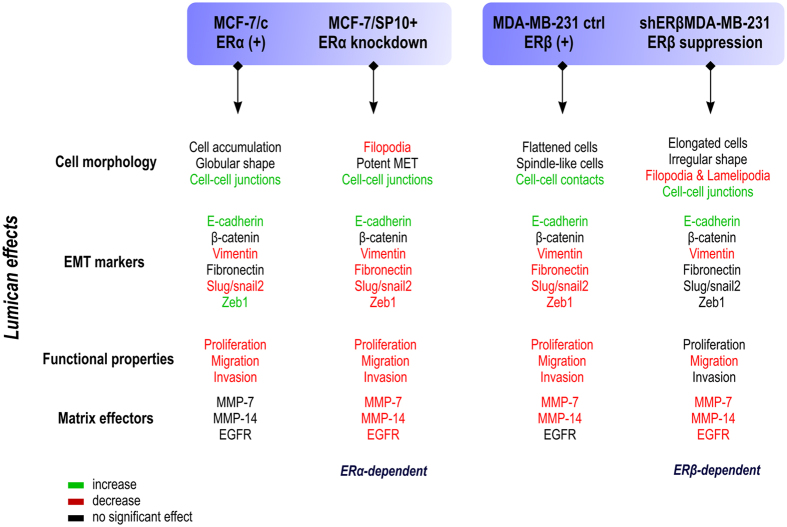
Diagram summarising the main functions of lumican in breast cancer cells. The actions of lumican have been classified according to its effects on cell morphology, epithelial-to-mesenchymal transition, functional properties (proliferation, migration and invasion), expression of matrix metalloproteinases and EGFR, according to the expression of estrogen receptors alpha and beta.

Notably, lumican inhibits the gene expression of pro-EMT transcription factors slug/snail-2 and zeb-1 in MCF-7/SP10+ cells. Zeb-1 is described to be a transcriptional activator in aggressive cancer types^29^,^30^. The above data are in agreement with the anti-migratory and anti-invasive properties of lumican. Regarding MDA-MB-231 cells, the obtained data confirmed the spindle-like appearance, the absence of E-cadherin, and the clear invasive phenotype, whereas shERβMDA-MB-231 cells exhibited an increased number of cell-cell contacts, and a tendency to regain the epithelial phenotype. Lumican-treated MDA-MB-231 cells appear more flattened and grouped, exhibit a significant increase in the I/L ratio and cell-cell contacts. These data are in agreement with the significant inhibitory effect of lumican in the invasion of MDA-MB-231 cells. Regarding the lumican-treated shERβMDA-MB-231, they appear as epithelial like cells with increased I/L ratio, and decrease in the number of lamellipodia and filopodia. It is noticing that the effect of lumican is inhibitory not only in the mesenchymal, by nature, aggressive cells MDA-MB-231, but even in the in the MCF-7/SP10+, which were modified in mesenchymal phenotype after the suppression of ERα. It is worth mentioning that the emerging result from this study is the participation of ERs, which is of vital significance in certain functional properties, such as cellular proliferation. Additionally, the considerable decrease of the invasive capacity of these cancer cells, can be correlated with the inhibition of the expression and activity of molecules, which are involved in the metastatic procedure of cancer. Taking into consideration the above data, it is suggesting that lumican is a powerful anticancer effector in terms of alteration of cancer cell properties and morphology affecting the EMT/MET process especially in the highly invasive MCF-7/SP10+ and MDA-MB-231 cells. All alterations induced by lumican, dependent or not from the ER status, in respects to cell morphology, gene expression of EMT markers, functional properties and matrix effectors are gathered in Fig. 7.

MMPs play key roles in physiological ECM remodelling, but also in cancer progression as they are implicated in invasion and metastasis^31^. MMP-14 plays critical role in cell migration, through regulation of the activity or expression of other MMPs, as well as activating other molecules implicated in cell migration, such as integrins. Our observations advocate that the lumican-induced suppression of the expression of MMPs, and mostly of MMP-14, comes in agreement with its inhibitory effect in breast cancer cells migration. Moreover, lumican achieved a striking decrease of the proteolytic activity of MMP-14 both in the highly-invasive MCF-7/SP10+ and in the aggressive MDA-MB-231 cells, rendering lumican as a critical inhibitor of breast cancer invasion and metastasis. This decrease of the significantly induced transmembrane MMP-14 by lumican in MCF-7/SP10+ could possibly explain the decreased level of invasion in the MCF-7/SP10+ cells treated with lumican, since a prerequisite for the invasive procedure of cells, is the localisation of MMP-14 within the filopodia. It was previously established that the altered activity of MMP-14 can serve as a potential mechanism of action of the anti-tumourigenic lumican^16^,^32^,^33^. Specifically, it was proven that the cell migration of the EMT-like B16F1 cells, induced by elevated level of Snail expression, was altered by lumican^34^. This proposed mechanism can be even more complex, as ΜΜP-14 affects cell migration, not only by adjusting the activity of downstream MMPs, but also by rendering active migration-implicated molecules, such as integrins and many related intracellular signalling pathways. The direct synergism of membrane MMPs, and especially of MMP-14 and integrins, was previously reported as a hallmark in tumour invasion and angiogenesis^34^,^35^. On the other hand, MMP-7 has been related with disease progression and its expression has been reported to be mediated by TGFβ^36^. Considering the critical role of TGFβ in the EMT process, the inhibitory effect of lumican on MMP-7 may also well be related to its effect on cell morphology.

In this study, it has been shown for the first time in the literature that lumican plays an important anticancer role in the transformation of epithelial cells in mesenchymal phenotype, which constitutes a key factor in the metastasis of cancer. Besides the existing reports concerning the anticancer effect of lumican, the mechanism of action has not been fully elucidated. The effects of lumican discussed up to date may be due to different levels of action, concerning either its interactions with ECM molecules or intermediation in the activity of membrane receptors^16^,^27^.

The expression of lumican in human breast carcinoma was first reported by Leygue and collaborators^24^. Lumican was shown to be the most abundant proteoglycans in breast tumours. Lumican and decorin appeared to be inversely regulated in association with breast cancer tumourigenesis. Interestingly, high level of lumican expression in breast cancer tissue was shown to be correlated with low level of expression of ER in the tumour. Although this specific study was rather small in terms of number of patients, it would be interesting to study whether lumican endocytosis might occur and might be regulated either directly or indirectly by the level of expression of ER at the cell membrane. In both cases, lumican effect on EMT/MET transition of breast carcinoma cells might be partly explained by an endocytosis mechanism either of lumican, ER or by an alteration of their expression. In a larger cohort of patients, a more recent study showed that a reduced expression of lumican and decorin is associated with poor outcome of invasive breast cancer including ERα-negative status^37^. Thus, the epithelial phenotype induced by lumican in ERα-negative invasive cells (MCF-7/SP10+) might suggest that a lumican-based treatment of these patients would be beneficial in terms of survival since it inhibits EMT. However, further works are necessary to better understand the lumican mechanism of action in EMT/MET transition. Actually, lumican was shown to promote EMT in lung through the activation of the ERK 1/2 pathways^38^, whereas Wu and collaborators suggested an inhibitory role of the EMT by lumican^40^. On the other hand, overexpression of EGFR promotes migration and invasion of highly invasive cells, like MDA-MB-231 and MCF-7/SP10+^5^. Although the effects of the same SLRP family, decorin and biglycan, on the endocytosis of EGFR have been extensively studied^39^,^41^, lumican and EGFR interaction was not studied to our knowledge. Similar endocytosis pathways cannot be excluded as a mechanism of the regulation of the lumican effect on EMT/MET process. A quantitative analysis of type I collagen fibril regulation by lumican and decorin by Atomic Force Microscopy was reported^42^. The results showed that lumican or decorin core proteins alone have a strong potential to regulate the spacing and connectivity of collagen I fibrils.

Regarding the regulation of cell migration by lumican it has been reported that it is exerted via alteration of actin network and focal adhesions and is mediated by α2β1 integrin^19^,^43^. Integrins, regarded as the major receptors for the attachment of cells to ECM, are implicated in cell adhesion and migration. Lumican interacts directly with α2β1^44^. Interestingly, E-cadherin was described to be a ligand for integrin α2β1^45^. Moreover, since lumican was shown to interact directly with the α2 integrin subunit^44^, further investigations will be necessary to elucidate whether lumican and the monomeric form of E-cadherin compete to interact with α2 subunit during the EMT/MET process. One more mechanistic approach, including an in-/out-signalling cascade, was reported concerning lumican, secreted by human osteosarcoma cell lines. Lumican inhibited osteosarcoma cell adhesion through endogenous inhibition of TGFβ2. The altered expression of TGFβ2 activity, induced and downstream modulated the cascade of pSmad2, upregulated integrin β1, and was inversely associated with pFAK^46^. This potential effect of lumican on TGF-β signalling as a possible effector of EMT/MET would be of importance to be evaluated in breast cancer cells.

Our data point to the conversion of established EMT status of the aggressive breast cancer cells MCF-7/SP10+ and MDA-MB-231 into a more epithelial-like state, triggered by the treatment of cells with lumican and the knockdown of ERα and the suppression of ERβ, respectively. This EMT/MET reprogramming is accompanied by dynamic changes in morphology, in expression of matrix effectors and in alteration of cell functions, resulting in a less aggressive and less metastatic cell. The obtained data suggest that the treatment with lumican may be beneficial for the breast cancers and further studies on the mechanisms and particularly on the cell signalling affected in relation to the ER status will improve our understanding on the pathways affected.

## Materials and Methods

### Reagents

Recombinant human lumican (57 kDa) was purchased from R&D Systems (#2846-LU-050, R&D Systems, MN, USA). Recombinant human pro-MMP-14 (catalytic domain, amino acids 89-265) was obtained from Merck Millipore (Nottingham, UK). Prior to enzymatic activity assays, pro-MMP14 was incubated with APMA (AnaSpec, San Jose, USA) to convert the enzyme in its active form. Alexa-Fluor 568-labelled phalloidin was used for the staining of actin in immunofluorescence, and was purchased from Invitrogen Corporation, Carlsbad, USA. Prolong^®^ Gold antifade reagent with DAPI was supplied by life Technologies. Lumican was used in all experiments was added in serum free medium, at final concentration of 100 nM.

### Cell culture

MCF-7/c, MCF-7/SP10+, MDA-MB-231 ctrl and shERβMDA-MB-231 breast cancer cells were cultivated daily in liquified air 95% O_2_/5% CO_2_ at 37 °C and in DMEM medium, completed with 10% foetal bovine serum (FBS), 1.0 mM pyruvic acid, 2 mM L-glutamine and a cocktail of antibiotics (100 μg/ml of penicillin, 100 μg/ml of gentamicin, 2.5 μg/ml of amphotericin B). Cells passed with 0.05% (w/v) trypsin in PBS, containing 0.02% (w/v) Na_2_EDTA. Infections of MCF-7/c cells with shRNA against human ERα or the non-targeted shRNA control and respectively infection against the human gene ERβ for the MDA-MB-231 ctrl, was achieved with the use of Polybrene solution (sc-134220, Santa Cruz Biotechnology, Inc), following the instructions of the manufacturer. For the establishment of the stable clones, puromycin dihydrochloride (0.8 μg/ml) (sc-108071, Santa Cruz Biotechnology, Inc, USA) was added in the medium, and was replaced with fresh puromycin every 3–4 days. DMEM, serum, pyruvate acid, L-glutamine, penicillin, streptomycin, amphotericin B, and gentamicin were supplied by BioseraLTD (Courtaboeuf, France) as previously described^4^,^5^.

### Phase-contrast microscopy

Cells were cultivated upon 80% confluence, and they were starved for 16h. When needed, lumican was added in serum free conditions. The pictures of live cells on the well-plate, were selected by OLYMPUS CKX41 microscopy, equipped with digital camera CMOS color (SC30).

### Scanning laser confocal microscopy

Breast cancer cells were seeded (n = 50.000 cells/well) in a 24-well plate on sterile 12 mm in diameter glass coverslips. MCF-7/c, MCF-7/SP10+, MDA-MB-231 ctrl and shERβMDA-MB-231 and were grown up to 80% confluence on coverslips for 24 h. Non-coated coverlips were used as controls. Cells were fixed in ice-cold 4% paraformaldehyde. Cells were permeabilised with 0.1% Triton X-100 and incubated at 4 °C. E-cadherin, vimentin, β-catenin were immunodetected using the primary antibodies in dilutions, 1:100, 1:200, 1:100 respectively. Negative controls were performed using non-immune IgG or by omission of the primary antibody. The secondary Alexa Fluor^®^ 488-conjugated antibodies were used at a dilution of 1:200. Alexa Fluor^®^ 568-conjugated phalloidin was used to label filamentous actin. The antibodies are referred in Supplementary Table S1. Slides were observed under confocal laser scanning microscope: LSM 710 NLO (Zeiss, oberkochen, Germany).

### Ratio l/L

The ratio l (width)/L (length) of the cells was measured in order to clarify the number of globular, ovoid cells (epithelial phenotype) and the number of elongated, spindle-like cells (mesenchymal phenotype). The ratios presented are representative from an average of three different photos from every cell line and every photo included minimum ten cells. The measurement was carried out with ImageJ.

### SEM imaging

MCF-7/c, MCF-7/SP10+, MDA-MB-231 ctrl and shERβMDA-MB-231 breast cancer cells seeded in culture flasks were firstly rinsed with a phosphate buffer solution to prevent cells detachment and then fixed in a Karnovsky’s solution for 20 min. Flasks with adhering cells were again rinsed three times with 0.1% cacodylate buffer, postfixed in 1% OsO_4_ in cacodylate buffer for 20 min, dehydrated with increasing concentrations of ethanol, and finally dehydrated with hexamethyldisilazane (Sigma-Aldrich Inc) for 15 min. The specimens were mounted on appropriate stubs, coated with a 5 nm palladium gold film (Emitech 550 sputter-coater) to be observed under a SEM (Philips 515, Eindhoven, The Netherlands) operating in secondary-electron mode.

### Cell Proliferation Assay

Cell proliferation analysis was achieved through a colorimetric method, using WST-1 dye. Breast cancer cells were cultivated in a 96-well plate in medium in the presence of bovine serum, in a 7.500 cells/well density for MCF-7/c and 5.000 cells/well for MCF-7/SP10+, MDA-MB-231 control (ctrl), shERβΜDA-MB-231. After 24 and 48 h, lumican was added accordingly to the premix WST-1 reagent (soluble tetrazolium salt) in a 1/10 ratio. The absorbance was measured in 450 nm after 30 min, 1, 2 hours (reference wavelength: 650 nm). The dye was supplied by Takara Bio Inc., Japan.

### Migration Assay

Cells were seeded on 24-well plate in culture inserts (Biovalley, Marne-la-Vallee) at a concentration of 2 * 10^5^ cells/ml (70 μl of cell suspension per chamber). After 24 h of incubation, the culture inserts were removed, two washes with PBS followed, and the wells were filled with 2 ml of serum-free cell culture medium. When needed, 100 nM lumican (57 kDa) was added into the medium. The wound healing was observed at 0, 24 and 48 h, using an inverted microscope (Axiovert 200M; Zeiss, Oberkoken, Germany) equipped with a digital camera. Wound closure was estimated from 3 independent experiments by taking pictures of 3 microscopic fields (magnification 10x) per insert, 3 replicate inserts for each condition. The average wound area was measured by Image J software.

### Invasion assay

Twenty four- well plates were used and ThinCert™ inserts were added (24-well, pore size 8 μm, supplied by GreinerBio- One, Courtaboeuf, France). Fifty thousand cells were seeded in 200 μl of medium, containing 0.5% bovine serum albumin (BSA). When needed, 100 nM lumican (57 kDa) was added in the upper chamber exactly at the same time that cells were plated. The inserts were pre-coated with 50 μg of Matrigel^TM^, supplied by BD Biosciences, incubated at 37 °C for 1 hour. In the lower chamber, 800 μl of medium was added, containing 10% BSA, which acts as chemotactic factors for the cells. Medium containing 2% BSA in two different wells was used as a negative control. After 48 h of incubation, cells that did not achieve to permeate the Matrigel^TM^, and finally migrate, were removed from the upper side of the chamber with a cotton stick. The lower side of the chamber was chemically fixed with 4% paraformaldehyde for 20 min at room temperature.

For the exact determination of cells, chambers were stained with crystal violet for 10 minutes (staining of the nucleus), and afterwards, photos were captured with the use of an inverted microscope at a 10x magnification, equipped with a digital camera. Every experiment was confirmed 3 times, containing every sample in triplicate (n = 3) and every well was captured at least at 3 different areas.

### RNA isolation and Real-time PCR analysis

Breast cancer cells were grown in serum-containing medium up to 70–80% confluence. Cells were serum starved overnight. Afterwards, lumican as tested agent was added according to the experimental plan in serum- free culture medium for 24 h. Total RNA was isolated from cells using a NucleoSpin^®^ RNA II Kit (Macherey-Nagel, Duren, Germany). The amount of isolated RNA was quantified by measuring its absorbance at 260 nm. Total RNA was reverse transcribed using the PrimeScript 1st strand cDNA synthesis kit perfect real time (Takara Bio Inc., Japan) and KAPA Taq ReadyMix DNA Polymerase (KAPABIOSYSTEMS). Real-time PCR analysis was conducted in 20 μL reaction mixture, according to the manufacturer’s instructions. Relative expression of different gene transcripts was calculated by the ΔΔCt method. The Ct of any gene of interest was normalized to the Ct of the normalizer (GAPDH). Fold changes (arbitrary units) were determined as 2−ΔΔCt. Genes of interest and utilized primers are presented in Supplementary Table S2.

### Proteolytic activity of MMP-14

The activity of MMP-14 was measured in 96-well plate, using 1 μΜ of the fluorogenic substrate: 5-FAM/QXL520^TM^ of FRET peptide in reaction buffer, supplied by Sensolyte^®^ (AnaSpec, San Jose, USA) in wavelengths of excitation and emission, 490 nm and 520 nm respectively. First of all, MMP-14 was activated by using 1mM of APMA (4-amino-phenyl-mercuric acid) for 2 hours in 37 °C. All the samples were inserted in triplicate in the plate in 37 °C. The fluorescence was measured with a spectrofluorometer (Mithras LB940, Berthed Technologies, Thoiry, France). For the determination of the effect of the activity of MMP-14 *in vitro*, cells were cultivated with lumican for 48 h, and cell pellets were collected after trypsinisation and centrifugation.

### Statistical analysis

Results were expressed as mean ± SD. Statistical significance between groups was calculated using Student’s *t*-test. The *p* value ≤ 0.05 was considered statistically significant. Significance was also estimated with block two-way ANOVA, using the cell line and the presence of lumican as grouping variables, and the post hoc Scheffe’s multiple comparisons test and correct analysis. Asterisks (*), (**)indicate statistically significant differences (*p* ≤ 0.05 and *p* ≤ 0.01, respectively).

## Additional Information

**How to cite this article:** Karamanou, K. *et al*. Lumican effectively regulates the estrogen receptors-associated functional properties of breast cancer cells, expression of matrix effectors and epithelial-to-mesenchymal transition. *Sci. Rep.* **7**, 45138; doi: 10.1038/srep45138 (2017).

**Publisher's note:** Springer Nature remains neutral with regard to jurisdictional claims in published maps and institutional affiliations.

## Supplementary Material

Supplementary Data
