# Supplementary material for: Lumican effectively regulates the estrogen receptors-associated functional properties of breast cancer cells, expression of matrix effectors and epithelial-to-mesenchymal transition
Source: Sci Rep. 2017 Mar 23;7:45138. doi: 10.1038/srep45138 (PMC5362815; doi:10.1038/srep45138)
Supplement: Supplementary Data [file srep45138-s1.pdf]

Lumican effectively regulates the estrogen receptors-associated functional properties of breast cancer cells, expression of matrix effectors and epithelial-to-mesenchymal transition

Konstantina Karamanou, Marco Franchi, Zoi Piperigkou, Corinne Perreau, Francois- Xavier Maquart, Demitrios H. Vynios, Stéphane Brézillon

Supplementary data

Supplementary Table S1: List of antibodies used in this study.

Antibody	Manufacturer
anti-human vimentin, Clone RV202, monoclonal, mouse	BD, Becton & Dickinson Biosciences, Heidelberg, Germany
anti-human β-catenin, Clone 14, β-catenin, monoclonal, mouse	BD, Becton & Dickinson Biosciences, Heidelberg, Germany
anti-human E-cadherin, clone 36, monoclonal, mouse	BD, Becton & Dickinson Biosciences, Heidelberg, Germany
Alexa-Fluor 488, anti-mouse IgG, Goat	Life Technologies, Renfrew, UK

Supplementary Table S2: List of PCR primers used in this study.

Gene	Primer code / sequence	Primer type
MMP-7	Fwd: 5’- GCT GGC TCA TGC CTT TGC -3’<br>Rev: 5’- TCC TCA TCG AAG TGA GCA TCT C -3’	ABI TaqMan assay
MMP-14	Fwd: 5’- CAT GGG CAG CGA TGA AGT CT -3’<br>Rev: 5’- CCA GTA TTT GTT CCC CTT GTA GAA GTA -3’	ABI TaqMan assay
EGFR	Fwd: 5’ -ATG CTC TAC AAC CCC ACC AC -3’<br>Rev: 5’ -GCC CTT CGC ATC TCT TAC AC -3’	ABI TaqMan assay
E-cadherin	Fwd: 5’ -TAC GCC TGG GAC TCC ACC TA -3’<br>Rev: 5’ -CCA GAA ACG GAG GCC TGA T -3’	ABI TaqMan assay
Vimentin	Fwd: 5’ -GAG AAA TCC TGC TCT CCT CGC -3’<br>Rev: 5’ -GGC TCG TCA CCT TCG TGA AT -3’	ABI TaqMan assay
Zeb-1	Fwd: 5’ -GAA AAT GAG CAA AAC CAT GAT CCT -3’<br>Rev: 5’ -CCC TGC CTC TGG TCC TCT TC -3’	ABI TaqMan assay

Fibronectin	Fwd: 5’ -CAT CGA GCG GAT CTG GCC C -3’<br>Rev: 5’ -GCA GCT GAC TCC GTT GCC CA -3’	ABI TaqMan assay
Snail-2/slug	Fwd: 5’ -AGA CCC TGG TTG CTT CAA GGA -3’<br>Rev: 5’ -CTC AGA TTT GAC TCT GCA AA -3’	ABI TaqMan assay
GAPDH	Fwd: 5’- ACG GAT TTG GTC GTA TTG GG -3’<br>Rev: 5’ -TGA TTT TGG AGG GAT CTC GC -3’	ABI TaqMan assay
